# Changes in Patient-Reported Self-Confidence and Self-Esteem Following Gingival Depigmentation: A Questionnaire-Based Pre-post Study

**DOI:** 10.7759/cureus.117109

**Published:** 2026-09-28

**Authors:** Manali Gupte, Jasuma Rai, Monali Shah, Riddhika R Kochar, Heet Gada, Sakshi R Shah

**Affiliations:** 1 Department of Periodontology, K M Shah Dental College and Hospital, Sumandeep Vidyapeeth Deemed to be University, Vadodara, IND; 2 Department of Periodontology, K M Shah Dental College and Hospital, Sumandeep VIdyapeeth Deemed to be University, Vadodara, IND

**Keywords:** depigmentation, gingival depigmentation, gingival hypermelanin pigmentation, self-confidence, self-esteem

## Abstract

Background and objective

A smile is an essential aspect of facial aesthetics, with the gingiva playing a vital role, as its color and pigmentation significantly impact the overall aesthetic appearance. Gingival pigmentation can shape an individual's view of gingival aesthetics, and when perceived as undesirable, it may affect psychosocial aspects such as self-esteem and self-confidence. This study aimed to assess the self-confidence and self-esteem levels prior to and following the depigmentation procedure, as well as their correlation with the clinical Dummett Oral Pigmentation Index (DOPI).

Materials and methods

Forty participants with gingival hyperpigmentation were selected from the outpatient department. A gingival depigmentation procedure was performed. An investigator-developed questionnaire with eight paired questions, each asked twice, was used to evaluate self-confidence and self-esteem levels before and one month after surgery and the association with the DOPI. Fisher's exact test and Pearson's chi-square test were utilized for statistical analysis.

Results

Before depigmentation, 82.5% of participants reported being stared at, while 100% experienced self-consciousness, hesitation about displaying their gums, and reduced confidence; 70% reported offensive remarks and related unhappiness. Following depigmentation, these concerns were reduced to 0%, with significant improvements in self-confidence and self-esteem (p < 0.001). A notable correlation was found between the clinical DOPI score and both smile confidence and levels of self-esteem (χ² = 80, df = 2, p < 0.001).

Conclusions

Greater emphasis should be placed on aesthetic procedures such as gingival depigmentation, as they considerably affect individuals' self-esteem and self-confidence. Advancements in such aesthetic treatments go beyond merely enhancing outer beauty; they play a decisive role in nurturing inner confidence and empowerment.

## Introduction

Facial appearance significantly impacts how we perceive others, with a smile being a key element of facial aesthetics [1]. The aesthetics of a smile depend on the balance between teeth and gingival tissues, which includes factors such as tooth features and the shape, visibility, and color of the gums. Therefore, differences in gingival pigmentation can affect the perceived attractiveness of a smile. The color of the gums is mainly influenced by blood flow, the thickness of the epithelial layer, keratinization, and natural pigments such as melanin, carotene, oxyhemoglobin, and reduced hemoglobin, with pigmentation appearing as diffuse or localized brown, light-brown, or dark patches [1]. Although gingival pigmentation is generally benign, when perceived as aesthetically undesirable, it may influence satisfaction with one’s smile and potentially affect self-perception, self-esteem, and self-confidence.

An individual’s perception of their gingival appearance can impact their awareness of how their smile is perceived by others, which may result in feelings of self-consciousness when smiling or displaying their gums. Concerns about dental aesthetics can affect psychosocial well-being, self-image, and dental self-confidence, especially when individuals consider their gingival appearance to be aesthetically unpleasing [1,2]. The visibility and appearance of the gums are crucial elements of smile aesthetics, and personal perception significantly influences the evaluation of a smile’s aesthetic appeal [3-5]. As a result, concerns about gingival aesthetics can affect social behavior and lead to changes in self-confidence and self-esteem [1,2].

To treat gingival hyperpigmentation, periodontal plastic surgical procedures, such as gingival depigmentation, are used as treatment modalities. Gingival depigmentation is a periodontal plastic surgical procedure in which gingival hyperpigmentation is eliminated or decreased using various techniques. However, evidence regarding the effects of gingival depigmentation on patient-reported self-confidence and self-esteem remains limited. Therefore, this study aimed to evaluate how aesthetic procedures, specifically gingival depigmentation, impact individuals’ self-esteem and self-confidence, while also correlating these effects with the clinical Dummett Oral Pigmentation Index (DOPI).

## Materials and methods

Study design

This prospective, single-arm, pre-post interventional study evaluated changes in self-confidence and self-esteem following gingival depigmentation. The surgical technique was selected according to routine clinical practice/operator preference and was not considered a study variable. The objective of the study was not to compare treatment modalities but to assess patient-reported outcomes following depigmentation and the association between clinical DOPI and self-confidence and self-esteem levels of patients. The research was carried out following the guidelines of the Declaration of Helsinki. All patients received thorough information that detailed the study's objectives, guaranteed anonymity, and confirmed that participation was completely voluntary, and they provided written informed consent for the study. Before the commencement of the study, approval was obtained from the Institutional Ethics Committee (SVIEC/ON/DENT/SRP/Feb/24/59).

Sample size estimation

The sample was estimated using G Power software (3.1, Heinrich Heine University, Düsseldorf, Germany), based on an anticipated effect size of 0.8, α = 0.05, and 80% power for statistical analysis. The minimum required sample size was 34 participants. Allowing for approximately 20% attrition, 40 participants were recruited.

Description of the population

Hypermelanin gingival pigmented patients with a score of 1 to 3, according to the original DOPI [6], were included, and they were periodontally and systemically healthy, aged 18 to 36 years. The study did not include smokers or people with gingival pigmentation associated with syndromes, pregnant and lactating women, medically compromised patients, and those not willing to take part in the study.

Periodontal therapy

Each participant was recruited from the Outpatient Department of Periodontology, and they received initial periodontal treatment, which comprised oral hygiene instructions, plaque control, and prophylactic scaling. Gingival depigmentation was performed using the clinically indicated technique [conventional scalpel (20 participants) and diode laser (20 participants)]. DOPI score was measured at baseline and one month after the surgery. The surgical procedure began with local infiltration of an anesthetic agent. The depigmentation was executed by means of either conventional scalpel and laser technique, using a No. 15 surgical blade with a Bard-Parker handle (Aspen Surgical, Caledonia, MI) for scraping the pigmented epithelial layer.

The raw surface was completely irrigated with normal saline. Before starting the procedure, laser protective eyeglasses were offered to the operating staff and patients. In the case of the diode laser, a fibre optic delivery system and an 810 nm wavelength were used in the contact mode. The operated area was covered with periodontal dressing for seven days, and postoperative instructions were delivered [7]. All gingival depigmentation surgeries were performed by the same surgeon. Since the purpose of the study was to evaluate patient-reported outcomes following depigmentation, the type of surgical technique was not included as an independent variable in the analysis.

Questionnaire

A self-made questionnaire was devised containing eight questions, each asked twice, which measured preoperative and postoperative self-confidence and self-esteem levels. Content validity of the questions was ensured by distributing the questions to five subject experts (not included in the analysis) through printed hard copy. Scoring criteria for the questionnaire were as follows: Essential (1), Useful but not essential (2), and Not necessary (3). The Item-Level Content Validity Index (I-CVI) [8] was 0.94. An I-CVI of 0.94 is regarded as a strong indicator of content validity, which is essential for the validity of the research results. Hence, perfect agreement was obtained from all the experts, and therefore, all items were considered valid.

The questions were in three languages (Hindi, Gujarati, English). The questionnaire comprised two domains: Domain 1 consisted of demographic data, and Domain 2 included the perception of the individual’s own consciousness towards pigmentation and the perception of the individual due to social impact for the observation of pigmentation. The assessments evaluating self-confidence and self-esteem were carried out before and again one month after the depigmentation surgery. The questionnaire was assessed by the treating surgeon.

Statistical analysis

Statistical analysis was performed using SPSS Statistics, Version 21, for Windows (IBM Corp., Armonk, NY). Fisher’s Exact test was used for category comparison, and Pearson’s chi-square test was used for associations between category variables. As this was a pre-post interventional questionnaire study, the patients undergoing gingival depigmentation with scalpel and laser were not assessed clinically. A p-value < 0.05 was considered significant, and a p-value < 0.001 was highly significant.

## Results

The study included all 40 participants, who were evaluated before and after surgery. Participants ranged in age from 18 to 36 years, with 18 years as the minimum age for inclusion. The mean (± SD) age was 26.83 (± 4.03) years. Male participants had a mean age of 26.87 ± 4.21 years, and female participants had a mean age of 26.76 ± 3.91 years. Analysis of the questionnaire responses showed that, before depigmentation, 82.5% of participants reported that people often stared at their hyperpigmented gingiva (Question 1). All participants (100%) reported holding back when smiling (Question 2), feeling conscious of others' opinions (Question 3), and feeling hesitant to show their gums in mirrors, photographs, or videos (Question 6). All of them also reported that gingival pigmentation affected their confidence (Question 8). Additionally, 70% of participants reported receiving offensive remarks about their gingiva (Question 4) and feeling unhappy because of such remarks (Question 5). After depigmentation, all participants responded negatively to Questions 1-6 and 8, while all participants continued to express a desire for pleasing gingiva (Question 7). Statistically significant differences were observed between preoperative and postoperative responses for Questions 1-6 and 8 (p < 0.001), whereas the difference for Question 7 was not statistically significant (p > 0.05) (Table 1, Figure 1).

**Table 1:** Participant responses The values are in n (%)

Sr. No.	Items/questions	Self-esteem and self-confidence levels before the depigmentation procedure		Self-esteem and self-confidence levels after the depigmentation procedure		P-value
		Yes	No	Yes	No	
Q1.	Do people often stare at your black gums?	33 (82.5%)	7 (17.5%)	0 (0.0%)	40 (100%)	< 0.001
Q2.	Do you hold back when you smile?	40 (100%)	0 (0.0%)	0 (0.0%)	40 (100%)	< 0.001
Q3.	Do you feel conscious of what others think when you smile?	40 (100%)	0 (0.0%)	0 (0.0%)	40 (100%)	< 0.001
Q4.	Do you get any offensive remarks regarding your black gums?	28(70%)	12 (30%)	0 (0.0%)	40 (100%)	< 0.001
Q5.	Do you feel unhappy when you get such remarks?	28(70%)	12 (30%)	0 (0.0%)	40 (100%)	< 0.001
Q6.	Do you feel hesitant to show your gums in the mirror, photographs, and videos?	40 (100%)	0 (0.0%)	0 (0.0%)	40 (100%)	< 0.001
Q7.	Do you wish your gums looked pleasing?	40 (100%)	0 (0.0%)	40 (100%)	0 (0.0%)	> 0.05
Q8.	Do your black gums affect your confidence level?	40 (100%)	0 (0.0%)	0 (0.0%)	40 (100%)	< 0.001

**Figure 1:**
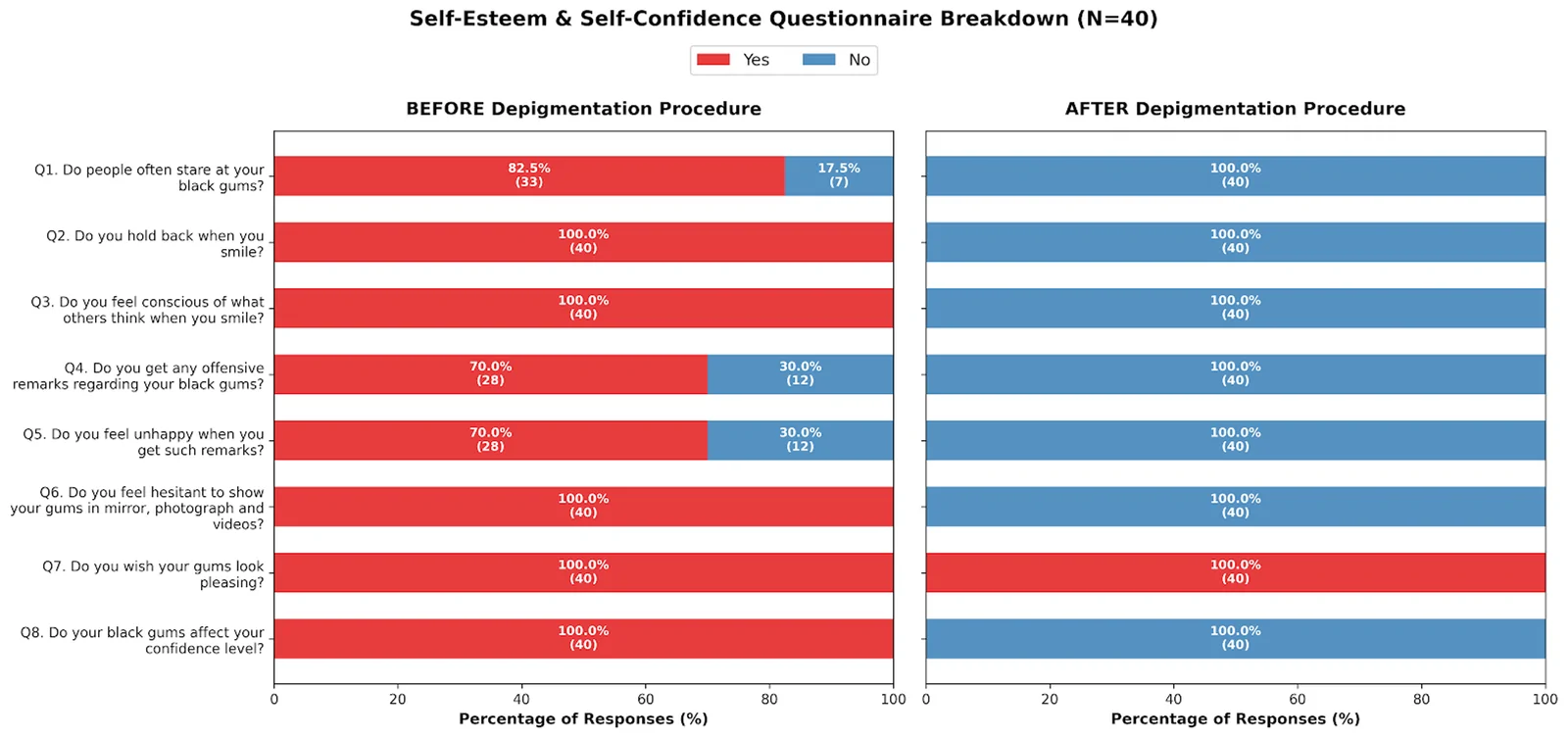
Graphic depiction of patient responses

Patient-reported outcomes showed a significant improvement in smile confidence and self-esteem following gingival depigmentation. Before treatment, all participants had gingival pigmentation, with 18 participants (45%) having a DOPI score of 2 and 22 participants (55%) having a DOPI score of 3. One month after treatment, all 40 participants (100%) achieved pink gingiva (no pigmentation) and reported confidence in their smile. A highly significant association was observed between the clinical DOPI score and levels of self-confidence and self-esteem, which shared a single unified value (χ² = 80, df = 2, p < 0.001).

## Discussion

This study aims to highlight the implications of aesthetic procedures, predominantly gingival depigmentation, in the general population. These procedures play a fundamental role in shaping individuals' levels of self-esteem and self-confidence, mainly because of their social influence and how individuals perceive themselves. The study population included individuals who displayed particular concern about their gums. Most were actively seeking job opportunities and looking for suitable partners, which indicates a phase in life in which personal appearance and social acceptance hold significant value.

Patient-reported outcomes showed a notable improvement in smile satisfaction after treatment [9], which highlights the significance of patient-centered care in periodontology. Although physiological gingival melanin pigmentation is harmless and is not a sign of disease, darker gingival pigmentation can be viewed as aesthetically unpleasing and may affect how attractive a smile is perceived to be [1]. In this study, depigmentation was linked to a substantial increase in self-confidence and self-esteem, likely reflecting greater satisfaction with gingival appearance after treatment. This observation is supported by Goswami et al. [10], who found that 75% of participants considered dark gingiva unattractive.

The smile is regarded as one of the most important facial expressions and plays a vital role in interpersonal communication, conveying emotions such as friendliness, acceptance, and appreciation [11]. Concerns about smile aesthetics can affect an individual's self-image and social interactions, especially when there are issues with the visibility or appearance of the gingiva. In this study, participants reported that they often restrained their smiles to hide their gingiva and felt self-conscious about how others perceived them, which implies that gingival pigmentation may have psychosocial effects that extend beyond its clinical appearance. After undergoing gingival depigmentation, a highly significant improvement was noted in the tendency to smile freely (p < 0.001), reflecting increased confidence in showing their smile. These results align with the findings of Ghani et al., who established a significant link between gingival features and smile attractiveness [11].

The current study demonstrated a significant association between gingival depigmentation and improved self-confidence and self-esteem. After treatment, participants expressed higher satisfaction with their smiles and a greater inclination to show their gingiva in mirrors, photographs, and videos (p < 0.001). Responses regarding the aspiration for aesthetically pleasing gingiva also showed a highly significant improvement (p < 0.001), which indicates increased satisfaction with the appearance of their gums after treatment. These results suggest that gingival depigmentation may provide benefits beyond aesthetic improvement alone by positively affecting patients' perceptions and confidence in showing their smiles. Earlier studies have mainly focused on pain and healing outcomes after depigmentation [12], and patient-reported psychological outcomes such as self-confidence and self-esteem have received limited exploration.

Limitations

This study has several limitations, including its small sample size (n = 40), absence of a control group, observational pre-post design, potential selection bias, and the fact that the subjects were young adults, which may restrict the applicability of the findings to older populations. In addition, needs and desires regarding appearance may vary across different life stages and circumstances. It must also be acknowledged that factors such as age, sex, and educational attainment can influence a person's satisfaction with their appearance [1]. The study used a self-made questionnaire to measure self-esteem and self-confidence before and after gingival depigmentation. Although it is useful for assessing patient perceptions, further validation in broader and more diverse populations would improve its applicability. Therefore, future studies should include larger, more representative samples, appropriate control groups, and validated patient-reported outcome measures.

## Conclusions

The findings of this study demonstrated a clear, measurable link between gingival depigmentation and improved levels of self-esteem and self-confidence. Gingival depigmentation may positively influence patients' self-perception, self-confidence, and satisfaction with their smiles. These benefits may extend beyond aesthetic improvement to psychosocial well-being and quality of life, which highlights the importance of incorporating patient-reported outcomes when evaluating the effectiveness of gingival depigmentation.

## Appendices

Questionnaire

Note: This questionnaire was administered before and after gingival depigmentation.

Questions on Concerns About Self-esteem

1. Do people often stare at your black gums?

Yes/No

2. Do you hold back when you smile?

Yes/No

3. Do you feel conscious of what others think when you smile?

Yes/No

4. Do you get any offensive remarks regarding your black gums?

Yes/No

Questions on Concerns About Self-confidence

5. Do you feel unhappy when you get such remarks?

Yes/No

6. Do you feel hesitant to show your gums in the mirror, photographs, and videos?

Yes/No

7. Do you wish your gums looked pleasing?

Yes/No

8. Do your black gums affect your confidence level?

Yes/No
